# EU-Funded Telemedicine Projects – Assessment of, and Lessons Learned From, in the Light of the SARS-CoV-2 Pandemic

**DOI:** 10.3389/fmed.2022.849998

**Published:** 2022-04-28

**Authors:** Laura Paleari, Virginia Malini, Gabriella Paoli, Stefano Scillieri, Claudia Bighin, Bernd Blobel, Mauro Giacomini

**Affiliations:** ^1^Research, Innovation and HTA Unit, A.Li.Sa.- Liguria Health Authority, Genoa, Italy; ^2^Department of Informatics, Bioengineering, Robotics and System Engineering, Genoa University, Genoa, Italy; ^3^Medical Oncology, IRCCS Ospedale Policlinico San Martino, Genoa, Italy; ^4^Medical Faculty, University of Regensburg, Regensburg, Germany

**Keywords:** tele-health, tele-care, telemedicine, tele-monitoring, tele-rehabilitation

## Abstract

The SARS-CoV-2 health emergency has demonstrated the need for developing structured telemedicine systems to protect citizens from the spread of the virus. Thereby, their importance and the necessity to tailor their diffusion at large scale for providing services both at a distance and in time has been shown. For these reasons, the European Union advocates the digital transition of health systems for the next 5 years. The main aim of this work is to revisit the telemedicine research projects financed by European Community during the period 2000-2020 with particular respect to the results derived from their application. The analysis showed that some integration of tele-care and tele-health could be obtained with tele-monitoring systems and the implementation of Electronic Personal Record (EPR). Furthermore, telemedicine allows enhancing health care in critical environments, to protect health and life of the most vulnerable patients, and to encourage cross-border dialogue. The criteria of “from distance” and “timely delivered” are granted, but the effectiveness of the overall offered services highly depends on the availability and the quality of the input data. Unfortunately, this remains a relevant problem in the SARS-CoV-2 pandemic.

## Telemedicine Definition and Application in Europe

Telemedicine concerns all health practices, provided remotely, considered as an innovative medical service in contrast to traditional face-to-face practice. It allows breaking down the geographical distances and aims at equalizing access to care using information and communication technologies (ICTs), thereby enabling the secure transmission and sharing of medical data and information for monitoring and controlling patients' clinical status.

**Figure 1:**
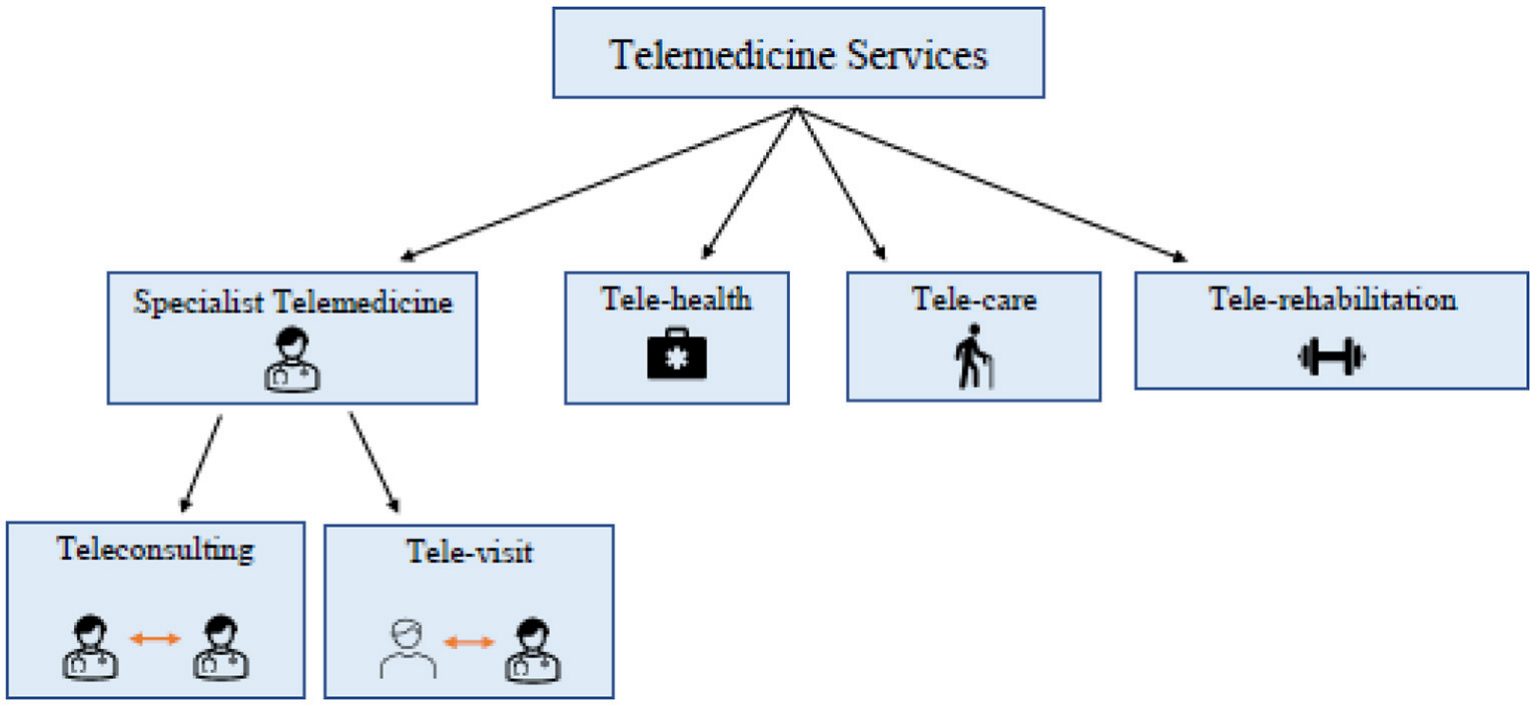
Schematic representation of the different telemedicine components.

Telemedicine services are classified as specialist telemedicine that comprises tele-visit and teleconsulting; tele-health; tele-care; and tele-rehabilitation. In particular, tele-visit is a medical action, which involves the health professional and the patient resulting in a remote electronic prescription of specialist visits or therapies. Teleconsulting, instead, is an information exchange activity between physicians and/or health workers on a specific clinical case for the provision of a second opinion. It plays a key role in emergency cases. Tele-health mainly concerns the management of patients with chronic diseases and allows general practitioners to monitor and manage them. Tele-care is related to the provision of health care services at citizens' home, especially addressing the elderly population. Tele-rehabilitation is a medical action that aims at recovering cognitive and physical performance status of patients. In addition, tele-monitoring can be considered as an operative procedure, which aims at the control of physiological parameters, such as insulin or blood pressure, through the use of wearable devices. Figure 1 represents the different components of telemedicine.

**Figure 2:**
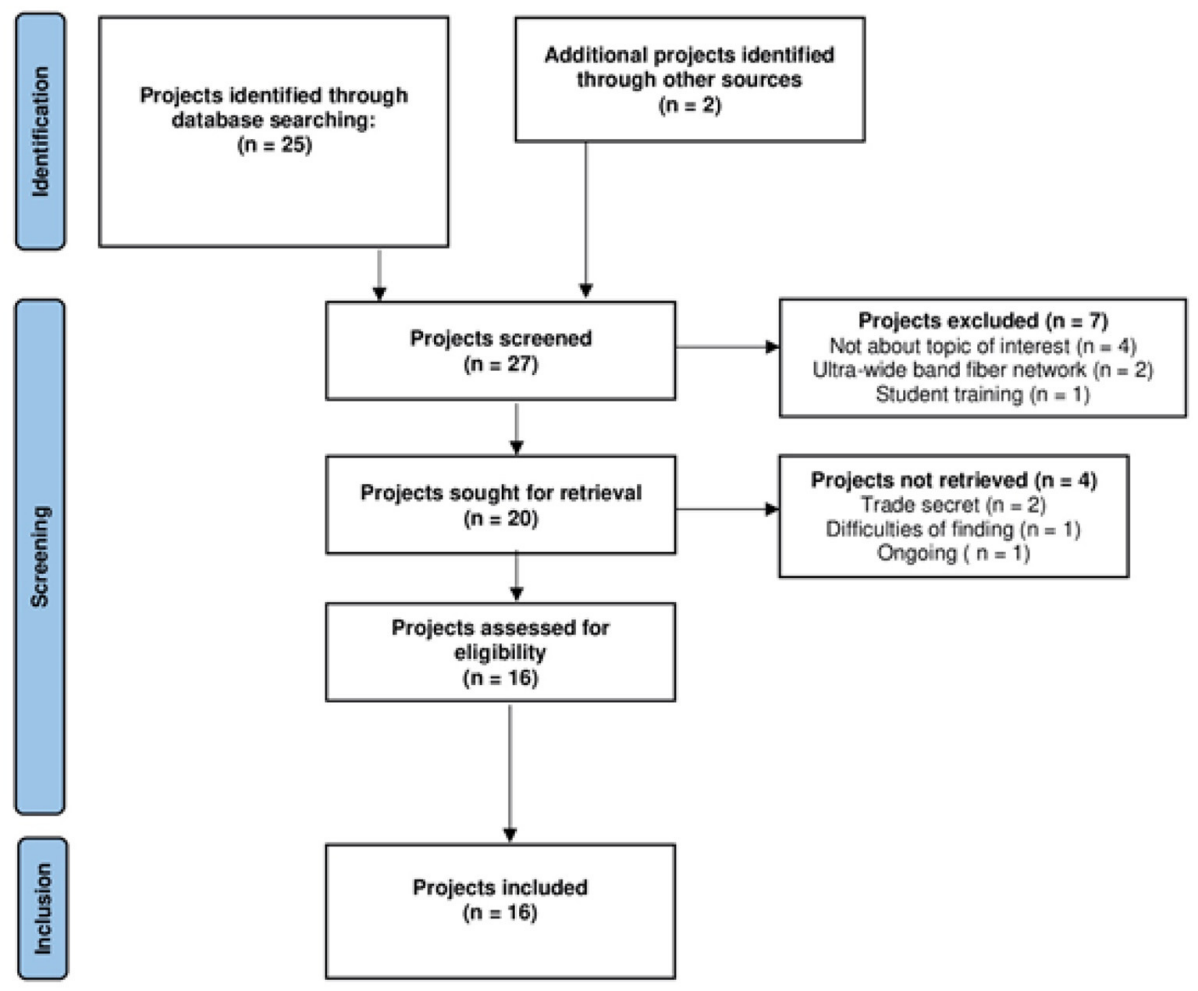
Flow diagram of project search and selection.

Throughout the last two decades, the European Community has been supporting telemedicine through the funding of several research projects powered by technological development and the consequent increase in interest in telemedicine. In fact, despite the opportunities and benefits related to Telemedicine services, to date their large-scale spread has been mostly slowed down by the high costs of technologies, the absence or inadequate laws for eHealth and privacy, the lack of capability to use ICT for elderly patients, the frequently unpredictable evolution speed of the patient status, but sometimes also the lack of qualified actors. Therefore, here we assess selected EU projects on telemedicine applications through the evaluation of their results to explore valid and persisting returns envisaged from their application/implementation. Figure 2 represents the flow diagram including the path we followed to select at least 20 projects. Table 1 shows the projects we have found by searching on https://ec.europa.eu/regional_policy/en/projects and https://artemis-ia.eu/projects-1.html, using the following keywords: “telemedicine,” “tele-health,” “tele-care,” “tele-monitoring,” “tele-rehabilitation” and “tele-visit” through the period 2000-2020. Moreover, we checked for reported project outcomes and the clinical area of intervention. Interestingly, almost all the selected projects applied telemedicine services to address general chronic disease management, and a few of them deployed telemedicine as a tool to diagnose and treat patients in a remote or rural areas. Chronic disease management continues to be one of the greatest healthcare challenges for providers. In fact, as chronic diseases continue to overstress health systems over time. Thus, telemedicine systems can improve efforts in chronic disease management, enhancing patients' engagement, improving the quality of care and the efficiency of used human and economic resources.

**Table 1:** Projects characteristics.

**Project name**	**Countries**	**Project duration**	**Medical field**	**Outcome**	**Reference**
TEL LAPPI Project	Finland	2000–2006	Radiology; Emergency system	Video conferencing, emergency care and electronic feedback system, as well as digital photographing and image transfer, were initialized in the Lapland Hospital District and member municipalities in the area near the hospital. Moreover, the long-term storage of images was readied and data protection was improved.	(1) https://ec.europa.eu/regional_policy/en/projects/Finland/telemedicine-services-in-lapland
IANUS	Spain	2007–2013	All medical fields	IANUS allows the recording of clinical data, this avoid of repeating procedures potentially harmful to patients. The most important result is the wide availability of clinical data for health care professionals. As a result, the gap between primary and secondary care is bridged.	(2) https://ec.europa.eu/regional_policy/en/projects/Spain/electronic-medical-record-system-ianus-improves-regional-health-care
The New Business Model for Ambulatory Monitoring of Patients Suffering from Congestive Heart Failure	Germany	2007–2013	Cardiology	The project has developed an outpatient model for the monitoring of chronic patients allowing the reduction of hospitalizations.	(3) https://ec.europa.eu/regional_policy/en/projects/Germany/high-tech-medicine-for-heart-patients-2
Telemedicine in the POMERANIA Euroregion	Germany Poland	2007–2013	Radiology; Oncology; Diabetology; Clinical pathology	Tele-tumor conferencing, Tele-radiology service and Tele-pathology service have been successfully established both in Germany and in Poland. Tele-ear nose throat service, Tele-ophthalmology service and Tele-stroke service have been only established in Germany. Data exchange between the two nations has improved.	(4) https://ec.europa.eu/regional_policy/en/projects/Poland/telepom-uses-ict-to-improve-medical-care-in-rural-areas-at-the-german-polish-border
The Telemedicine Pomerania project	Germany Poland	2010–2012			(4) https://ec.europa.eu/regional_policy/en/projects/Germany/telemedicine-pomerania-improves-healthcare-in-sparsely-populated-regions
The Competitive Health services project	Finland Partners: Ireland - Norway Scotland Sweden	2008–2010	Diabetology; Cardiology; Chronic patient with aphasia, dyslexia and Parkinson's disease	In Finland, the use of teleradiology has rapidly increased becoming common practice; three fourths of health centers used telelaboratory services and the coverage was 64% in 2007. Scotland has demonstrated to have a solid base of existing eHealth initiatives, both at national level and locally in the Highland region. In 2008, Sweden recorded 45 afoot e-health services, some based on tele-consultation and tele-monitoring. In Norway, telemedicine services were used for remote diagnosis and advices of treatment, second-opinion, communication between staff and access to radiology report system.	(5) https://ec.europa.eu/regional_policy/en/projects/Finland/healthcare-goes-electronic-under-northern-skies
ICT for Health	Belarus; Germany; Denmark; Estonia; Finland; Lithuania; Latvia; Norway; Poland; Russia; Sweden	2009–2012	Patients with chronic disease	Health technologies have allowed patients to improve the prevention and the treatment of their chronic diseases. Thanks to self-monitoring technologies patients have increased the responsibility for their own health.	(6) https://ec.europa.eu/regional_policy/en/projects/Belarus/ict-for-health-strengthening-social-capacities-for-the-use-of-e-health-technologies-by-the-aging-population
CHIRON - Cyclic and person- centric Health management: Integrated appRoach for hOme, mobile and clinical eNvironments	United Kingdom - Slovenia - The Netherlands - Hungary - Belgium - Greece - Italy - Spain	2010–2013	Cardiology	This project has developed a prototype of middleware aimed at tele-monitoring chronic patients with congestive heart failure. It fully satisfies the requirements indicated by the personnel.	(7) https://artemis-ia.eu/project/17-chiron.html
Development Of Cross-Border Telediagnostic And Teleconsultation Network In Health Institutions (TELEDIAG)	Serbia - Romania	12/2010–02/2012	Radiology Oncology; Clinical pathology	The project has allowed creating a cross-border telediagnostic and teleconsultation network among 14 health units of the cross-border partners. Moreover, every health units have installed a software for telemedicine and specialized equipment.	(8) https://ec.europa.eu/regional_policy/en/projects/Romania/telediag-enables-faster-more-precise-diagnosis-and-better-treatment-for-patients-on-the-romanian-serbian-border
ITTS - Implementing transnational Telemedicine Solutions	Finland - Norway - Sweden - Ireland - Scotland	09/2011–03/2014	Diabetology Cardiology; Obese people; Patient with Inflammatory Bowel Disease; Patient with multimorbidity	ITTS project allowed implementing telemedicine solutions into everyday practice and the spread of knowledge among countries. The evaluation demonstrated that remote solutions are positively accepted by patients, there are positive returns on investment and the use of telemedicine can be sustainable.	(9) https://ec.europa.eu/regional_policy/en/projects/Finland/calm-cool-and-connected-telemedicine-project-boosts-health-care-in-the-northern-periphery
iAge: e-inclusion in Aging Europe	Netherlands Scotland Norway Germany Belgium Denmark	2012–2014	Elderly people	This project has enabled the development of the Home Automation Living Platform (HALP), which ensures elderly patients to stay alone in their houses as long as possible.	(10) https://ec.europa.eu/regional_policy/en/projects/Belgium/boosting-the-economic-and-social-e-inclusion-of-the-growing-over-65-group
SmartCare	Italy	2013–2016	Cardiology	SmartCare project has allowed reducing days of hospitalization for chronic patients, mainly for those with heart failure. Moreover, it has ensured a sustainable use of local nursing resources.	(11) https://ec.europa.eu/regional_policy/en/projects/Italy/smartcare-using-ict-to-enable-older-people-to-live-independently-for-longer
SOS MAM	France Switzerland	2013–2015	Patients with acute mountain sickness – mountain tourists – mountain dwellers	The project allowed setting up a telemedicine call center for expedition teams. Currently 10 medicine doctors work through an association called Altidoc to maintain telemedicine system. Through Altidoc they provide teleconsultations before departure and telemedical guidance to groups during their expeditions. The follow-up project is called e-Res@MONT.	(12) https://ec.europa.eu/regional_policy/en/projects/France/sos-mam-telemedicine-for-a-mountain-environment
GAPP - Gamification Against Phantom Pain	Germany	2015–2018	Patients with phantom limb pain	The project has allowed creating a tele rehabilitation platform for patients with phantom limb pain.	(13) https://ec.europa.eu/regional_policy/en/projects/Germany/gapp-german-project-treats-phantom-limb-pain-with-the-help-of-virtual-reality
Beratung zum Eintritt in den Gesundheitsmarkt in den USA	Germany	2015–2016	Diabetology	ESYSTA® system has simplified interaction and communication between doctors and patients, has significantly improved blood glucose control due to the optimized self-management by the patients and has simplified the documentation process for patients.	(14) https://ec.europa.eu/regional_policy/en/projects/Germany/advice-on-entry-into-the-us-healthcare-market
e-Res@MONT	Lead Partner: France Partners: Italy Switzerland	2016–2018	Patients with acute mountain sickness – mountain tourists – mountain dwellers	The e-Res@MONT teleconsultation platform reduces the distance between patients and medical staff in a challenging environment, and improves the timeliness of monitoring, diagnosis and treatment. The telemedicine platform can also be used for medical tourism while removing language and cultural barriers.	(12) https://ec.europa.eu/regional_policy/en/projects/France/making-it-work-for-mountain-medicine-services-around-mont-blanc
E-coordination of bone and joint care in Cher	France	2016–2018	N/A	N/A	https://ec.europa.eu/regional_policy/en/projects/France/a-telemedicine-platform-facilitates-access-to-bone-and-joint-care-in-the-center-val-de-loire-region
AIR CARDIO	Italy	2017–2020	Cardiology	The AIR CARDIO project has developed a digital platform, which allows doctors to monitor the health of children suffering from congenital heart disease. During the Covid-19 health emergency, this system has allowed patients to decrease the risk of infection.	(15) https://ec.europa.eu/regional_policy/en/projects/Italy/air-cardio-a-lifeline-for-babies-with-heart-disease-in-tuscany
Community Areas of Sustainable Care and Dementia Excellence in Europe (CASCADE)	Lead Partner: France Partners: Belgium - Netherlands - United Kingdom	2017–2021	Elderly people and people living with dementia	CASCADE has developed a sustainable approach to elderly/dementia care, which has also reduced the strain on hospital beds and increased the quality of care. CASCADE allows people living with dementia to stay in their homes for as long as possible.	(16) https://ec.europa.eu/regional_policy/en/projects/Belgium/cross-border-project-develops-holistic-approach-to-dementia-care
Moore4Medical	Switzerland Germany Ireland Hungary Italy Spain Austria Netherlands Finland	2020–2023	People with sleep disease.	Ongoing project that aims at creating a bed-monitoring platform to control indiscreetly patients during their daily life.	(17) https://artemis-ia.eu/project/198-Moore4Medical.html

## The Coronavirus Lesson for Healthcare Transformation

The Coronavirus has confronted almost all countries around the globe with a series of unprecedented health, social, ethical and economic challenges. The pandemic has brought to light the consequences of a series of old problems that have exacerbated numerous situations of vulnerability, marginalization and suffering. In Italy, the pandemic has violently hit the most vulnerable people, while worsening the significant inequalities that plague our country, as evidenced by the social differentials that can be found in the excess mortality caused by COVID-19 (18). The health emergency has highlighted the strengths and criticalities of the sanitary system. The austerity policies adopted during the years have made it more efficient, but unprepared to deal with one demand shock like that imposed by the pandemic. The territorial services failed to stem the emergency in a timely manner. The hospitals challenged with COVID-19 cases have proved to be in difficulty in dealing with a pressure, due to the constant dwindling of economic resources, health personnel, and beds, which have been shorten over the last decades. In Italy, the protraction of expenditure control of health services recorded between 2009 and 2018 a particularly large reduction of the resources allocated to health, which has extended the gaps in terms of public health expenditure per capita. In 2018, the expenditure per capita was in Germany twice as much, and France 60 percent higher, than the Italian one (19).

### Government Responses

In order to support the recovery and resilience of Member States, the European Union approved the Next Generation EU program (20, 21), which allocates 750 billion € (20–22). One of the aims of this ambitious project, which will have closed by 2026, was to improve the digital transition of healthcare systems (20–22). Italy is the first beneficiary of this innovation program, because it was the most affected Member State, and it approved its National Recovery and Resilience Plan (21, 22). It promotes smart, sustainable and inclusive growth both through investments aimed at enhancing physical and human capital, and reforms, which should have an impact on productivity and competitiveness over the medium and long term. The European Union shall make available to Italy a financial contribution in the form of non-repayable support, to be legally committed by 31 December 2022. Moreover, the Mission 6.1 of the plan is focused on proximity networks, facilities and telemedicine for territorial healthcare assistance (22). The objective of this component is to strengthen the Italian NHS, enhancing the protection against environmental and climate-change related health risks, and better responding to the communities' needs regarding local care and assistance (22). In fact, local healthcare assistance is fragmented and subject to regional disparities that result in different levels of healthcare provisions and health outcomes across regions. The provision of integrated home care services is considered low, and the different healthcare and social service providers are considered to be only weakly integrated. The Investment 1.2 on Home as the first place of care and telemedicine consists in a large-scale adoption of telemedicine solutions and supporting healthcare innovation (22). The goal is to increase the number of people treated in home care to 10% of the population over 65 through investment in hardware and increased service provision, and the establishment of Territorial Coordination Centers (22).

The governance of the Italian Recovery and Resilience Plan is divided into several levels. The Ministry of Health is entrusted with the leadership of the project, together with the government working group, which must ensure that the execution is consistent with the political direction, the timing of the Plan and the needs of the territories (21, 22). AGENAS, the Italian National Agency for Regional Health Services, is responsible for implementing the Mission 6 (21), and its technical working group will be in charge to draw up the projects' guidelines, evaluate the proposals, oversee the regional procedures, and receive and verify the reports sent by the Regions.

**Figure 3:**
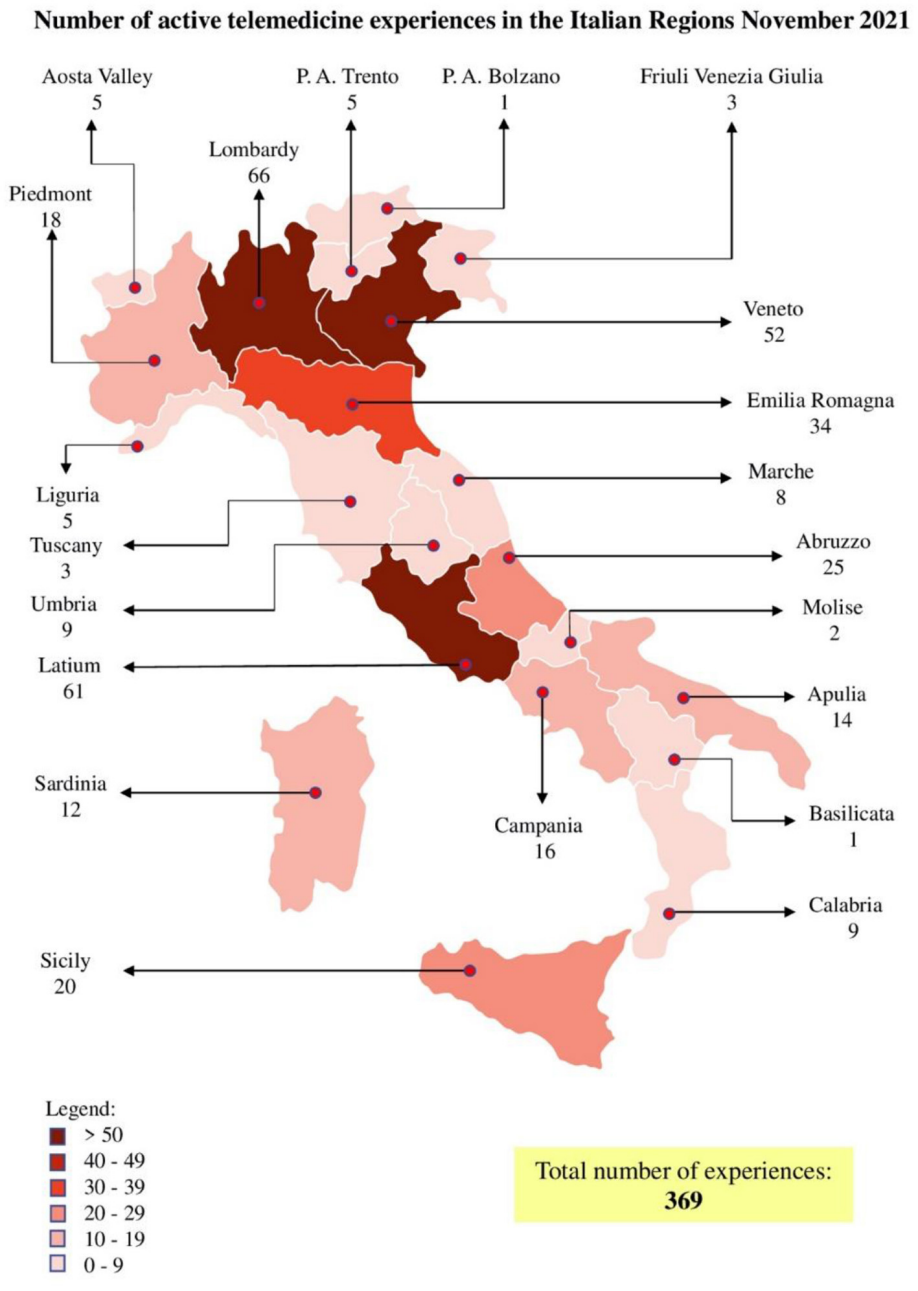
Distribution of active Telemedicine experiences in Italy.

As exposed above, the COVID-19 pandemic has highlighted the importance of technology, which allows developing structured and organized systems based on telemedicine services (23). At the outbreak of the COVID-19 pandemic in Italy, no appropriate rules within telemedicine have been implemented. The only one, dated in 2014 (24), was very vague and generic, limiting itself to the presentation of indications on the definitions, regulatory aspects and tariff services without a real boost to the application of telemedicine services. This caused the diffusion of local and isolated experiences. Indeed, each Italian Region has adopted protocols based on telemedicine services to delimit the virus spread, establishing the need

to define a national standard. In November 2021, the number of active telemedicine experiences in Italy were 369 and unequally spread at the national level as presented in Figure 3. Considering the Italian Regions, Liguria has implemented tele-visit through a dedicated platform and individual experiences of tele-monitoring and tele-consulting for cardiological, nephrological, and diabetic area, whilst there is no documented experience of tele-rehabilitation. Moreover, in our Region, the Policlinic Hospital San Martino has implemented telemedicine service for cancer patients, but only for cases that do not require a clinical visit but only a consultation to view laboratory or radiological tests performed externally.

## Specialist Telemedicine Projects

Among the projects selected for this study, 6 are focused on Specialist Telemedicine and briefly described below (Table 2). Since the early 2000's especially with Tel Lappi project, Finland has approved political strategies aimed at setting the foundations for a teleconsulting system through the acquisition of the equipment for videoconferencing for strengthening the emergency system in the Lapland Hospital District (1). Moreover, the health personnel involved in this project was trained to correctly use these technologies (1). As already stated, teleconsulting simplifies emergency procedures in problematic and rural areas. The SOS MAM project and the following e-Res@Mont project have developed a teleconsultation platform around the Mont Blanc thanks to the co-operation between France, Italy and Switzerland. This innovative platform permits nurses from mountain huts to exchange medical opinions with doctors based on the hospital in Aosta, the main city of the Region, during emergencies. Furthermore, to prevent potential connectivity problems, researchers have developed an offline application to support nurses' clinical evaluation when the connection is absent (12). Starting from 2002, the Pomerania Euroregion, the border area between Germany and Poland (4), was the protagonist of several European projects (i.e., The Telemedicine Pomerania project and Telemedicine in the POMERANIA Euroregion project), which aimed at implementing videoconferencing network between the 2 countries. Interestingly, since 2012 on the German side, a multidisciplinary tele-tumor conference takes place every week in several hospitals, while on the Polish side this program is not implemented (4). However, tele-conferencing for board meetings is successfully developed (4) to improve the co-operation and the medical information exchange between the two nations. The Development of Cross-Border Telediagnostic and Teleconsultation Network in Health Institutions (TELEDIAG) project is an example of cross-border teleconsultation network between Serbia and Romania (8). As stated by the project researchers, teleconsulting offers many advantages such as time-savings during emergency state (1, 12), because rescuers can execute medical procedures under the guidance of doctors who are hospital based. Moreover, it leads to an economic saving because some activities can be realized moving data and not people (1, 12), and reducing travels at all (1, 4).

**Table 2:** Projects classification.

**Specialist telemedicine**	**Tele-health**	**Tele-care**	**Tele-rehabilitation**
TEL LAPPI Project (1)	The Competitive Health services project (5)	Beratung zum Eintritt in den Gesundheitsmarkt in den USA (14)	GAPP - Gamification Phantom Pain (13)
SOS MAM (12)	ICT for Health (6)	SmartCare (11)	The Competitive Health services project (5)
e-Res@MONT (12)	The New Business Model for Ambulatory Monitoring of Patients Suffering from Congestive Heart Failure (3)	AIR CARDIO (15)	ITTS - Implementing transnational Telemedicine Solutions (9)
Telemedicine in the POMERANIA Euroregion (4)	IANUS (2)	iAge: e-inclusion in Aging Europe (10)	
The Telemedicine Pomerania project (4)	Community Areas of Sustainable Care and Dementia Excellence in Europe (CASCADE) (16)	The Competitive Health services project (5)	
Development Of Cross-Border Telediagnostic And Teleconsultation Network In Health Institutions (TELEDIAG) (8)	Beratung zum Eintritt in den Gesundheitsmarkt in den USA (14)	IANUS (2)	
	AIR CARDIO (15)	ITTS - Implementing transnational Telemedicine Solutions (9)	
		ICT for Health (6)	
		CHIRON - Cyclic and personcentric Health management: Integrated appRoach for hOme, mobile and clinical eNvironments (7)	
		Moore4Medical (17)	

## Tele-Rehabilitation Projects

In 2009, Scotland, Finland and Sweden have developed rudimental services based on ICTs to support rehabilitation for elderly and chronic patients with web services, audio and music programs, and videogames (5). Subsequently, the 9 remote exercise classes for rehabilitation of the ITTS (Implementing Transnational Telemedicine Solutions) project focused on the generation of a rehabilitation program. In this project, rehabilitation was based on videoconferences between physiotherapists and home-based patients, becoming a common medical practice in Scotland and Northern Ireland (9). The Gamification Against Phantom Pain (GAPP) project has set up a prototype of a tele-rehabilitation platform for patients with phantom limb pain. Thanks to this mobile platform, patients are able to exchange messages with therapists and select their training program centered on mirror therapy (13), which can be remotely executed during daily life practices. Moreover, this mobile application helps therapists to monitor and manage the phantom limb pain.

## Integration of Tele-Care and Tele-Health Projects

Tele-care integration can be realized through the installation of ICT solutions, such as sensors, automatic controllers, tele-alarm systems and portable devices (25), inside the homes, for instance, of elder, chronically ill, and disabled citizens. During the course of the iAge (e-inclusion in Aging Europe) project, it has been shown that most of the homes in the Netherlands are not suitable for disabled citizens due to uncomfortable bathrooms and narrow hallways (10). To overcome these architectural limitations, opened elevators, bathroom equipment and electronic doors could be installed (25). Furthermore, there are three projects centered on the implementation of tele-care solutions. The ITTS project has established 2 tele-care programs: one for chronic patients and the other for patients with multi-morbidity. This project aims at improving patients' independence through the installation of several technologies in their home, such as epilepsy and disability discrimination sensors (9). Another example of tele-care integration is represented by the iAge project, which has involved the North Sea Regions. This project encompasses pilot programs aiming at the creation of a comfortable home environment based on ICTs and home automation for the elderly population. This project has developed the Home Automation Living Platform (HALP) allowing elderly people to live safely alone in their houses. In fact, the system, through the use of sensors, is able to manage all the devices present in the house and detect eventual falls (10). Moreover, the Italian SmartCare project of tele-care integration supported by ICT is focused on elderly with chronic diseases with particular attention to heart failure. The results of the study show that the use of ICT can reduce the days of hospitalization due to the monitoring of patients' clinical parameters (26). Since 2006, in Scotland, the Scottish Center for Tele-health has been established to support the development of clinical tele-health projects. Additionally, in 2005, strategies to improve both tele-health and tele-care in the rural NHS Highland Area have been approved for supporting direct patient care, educational programs and videoconferencing networks (5). Besides, tele-health integration can be realized through the development of solutions that strengthen the integration between primary care and community services. These models are well represented by the Community Areas of Sustainable Care and Dementia Excellence in Europe (CASCADE) project, that provides flexible solution via the creation of residential facilities for demented and elderly patients (16). In addition, the New Business Model for Ambulatory Monitoring of Patients Suffering from Congestive Heart Failure project has developed a health care center based on telemedicine solutions (3). Importantly, it has been shown that the creation of these structures for chronically diseased citizens avoids the risk that non-acute patients occupy the wrong hospital bed (21).

ICT solutions enable the integration of tele-health and tele-care through tele-monitoring systems and Electronic Personal Record (EPR). Tele-monitoring system can improve health care through the use of appropriate platforms and medical devices, like blood pressure meter and pulse-oximeter (17), which are able to manage the safe transmission of patients' data to physicians for the control/treatment of chronic diseases. In 2008, Sweden established two ICT-based tele-monitoring services: the Care@Distance for patients with heart insufficiency and the Checkup-remote Monitoring of Physiological Parameters developed for those who have to check parameters frequently (11). Another example of tele-monitoring systems is the CHIRON prototype, which is composed of a home platform connected with medical devices and sensors, and the ICU client, which is a web application for personnel to check the patient's health status (7). Two European projects have developed tele-monitoring tools still available on the market. The first is the ESYSTA® system created by a German company that is a wireless system used to control blood glucose and insulin values in diabetic patients (14). The second is the AIR CARDIO platform realized by GPI Spa, which enables to manage children affected by congenital heart disease (15). These systems can simplify the communication between doctors and patients and improve the responsibility of patients (14) in the management of their illness. However, these benefits can only be achieved through a better acceptance of ICT solutions on the part of older citizens who show an aversion and unfamiliarity toward such technologies (21). For this reason, the ICT for Health in the Baltic See Region project has developed educational programs for chronic patients, in particular for the elderly, to improve their ICT skills, showing an increasing acceptance of e-Health among the users (6). Currently, the Moore4Medical project is developing a bed-monitoring platform using remote sensing of clinical parameters without direct contact of the device with the chronic patient. This innovative approach facilities monitoring of patients with sensitive skin (17).

As already mentioned, the EPR represents a digital archive of medical information which can be shared between different health service providers and patients (3), improving the organization of health systems. The ICT for Health project has established a multi-lingual EPR to support citizens, in particular chronically ill ones, in their travels abroad, and the sharing of medical document in the Baltic See Region (6). In Galicia, in 2013, 2,785,430 patients allowing the recording/consultation of clinical data (i.e., diagnostic images) and the e-prescription of medicine possessed the EPR-IANUS. The results of this project have highlighted how the usage of this system has improved the integration between primary and secondary care thanks to the accessibility of medical data to all healthcare professionals (6, 7, 11, 14, 15).

## Study Limitations

This study is mainly affected by two limitations: the trade secret and the difficulty of retrieving the material. With respect to the trade secret, the beneficiaries of the selected projects are companies. Thus, it was not possible to receive project details. This specifically concerns the German Getemed for The New Business Model, developer of the Ambulatory Monitoring of Patients Suffering from Congestive Heart Failure project, the German Emperra GmbH E-Health Technologies realizing the “Beratung zum Eintritt in den Gesundheitsmarkt in den USA” project, and the Italian GPI SpA with the AIR CARDIO project.

Through the trade secret approach, industries defend their products and knowledge, which cannot be handed over to others. The infraction of this privacy agreement classifies as crime. For this reason, the flow of information is reserved and confidential. The second limitation concerns the difficulty of finding the essential material for the studies' assessment because of the lack of project webpages and published documents. To solve this problem, we have tried to contact the different project referents or the beneficiary corporations resulting just in few replies. Moreover, it is important to point out that many selected projects took place several years ago, When the researchers of the contacted institutes were not working on it anymore, it leads to difficulties in finding the required materials. These limitations have restricted our study causing the inability in analyzing the outcomes of the projects and making it impossible to properly compare all the selected projects.

## Conclusions

In the present work, we have assessed 20 telemedicine projects developed in Europe during 2000-2020 to evaluate the strengths and the limits of these kind of emerging health services. Among them, several are based on teleconsulting: the Tel Lappi, SOS MAM, the e-Res@Mont, the Telemedicine Pomerania project, the Telemedicine in the POMERANIA Euroregion and the Development Of Cross-Border Telediagnostic and Teleconsultation Network In Health Institutions (TELEDIAG) (Table 2). With the exception of the first one, all the others are cross-border projects between neighboring countries. This aspect underlines the role played by teleconsulting, expandable to the telemedicine approach, to break down even the national borders, enabling the transparent spread of scientific knowledge and the co-operation among nations.

The development of home tele-care, which guarantees elder people to remain self-sufficient in their homes, has significant benefits such as cost savings for healthcare (13), because it allows the reduction of hospitalizations and the increase of their independence (10). It is important to know that for optimizing the realization of those e-Health approaches it is fundamental to identify, what the requirements and the needs of the final users are (10) in order to implement appropriate care models. For instance, the implementation of EPR also would ensure a better integration between hospitals and community services. A paradigmatic example is represented by the ICT for Health project that foresees a European portal for cross-border travel that would allow citizens, in particular chronic patients, to travel safely in European countries.

Through tele-monitoring, tele-health and tele-care technologies, the patients' responsibility on the management of their diseases can increase as their sense of safety (6). Moreover, tele-monitoring systems can enhance the healthcare for citizens who live in rural or problematic areas, enabling the continuity of care without patients' moving.

The Coronavirus has not only highlighted the criticalities of the health systems, but it has also pointed out the importance of telemedicine, which allows the reduction of waiting list, continuous monitoring, increasing the productivity of personnel, the reduction of traveling and the protection of patients from the spread of COVID-19. All these features make it possible to reorganize health systems, making them more sustainable and allowing economic saving. To face the health emergency, each Italian region has resorted to solutions based on telemedicine services, leading to the birth of many projects delivered irregularly (Figure 3). Such fragmentation is due to the lack of inter-regional dialogue, which makes standardization difficult (21). Currently, the Italian recovery and resilience plan is an essential occasion to enhance and homogenize telemedicine throughout the national territory. Another problem will be the certification of telemedicine activities and their correct storage, which allows personnel to recall the documents reported by facilitating territorial collaboration. The governance of telemedicine's diffusion will include the definition of technological and interoperability standards, national guidelines, and continuous check. Therefore, the Italian Recovery and Resilience Plan aimed at uniformly spreading telemedicine by promoting culture for appropriate and compassionate use.

In the last 20 years, ICTs have assumed an increasingly predominant role in health systems, allowing the integration of tele-care and tele-health, improving the lifestyle of citizens. Moreover, the pandemic emergency period has shown the centrality of health as a universal good and the fundamental importance of the NHSs, highlighting at the same time various areas on which to intervene. The historic opportunity now opens up to redefine the healthcare of tomorrow, with the obligation to make the best use of the incoming injection of economic resources derived from Europe. The challenge we face is to deal with the three fronts of acuity, chronicity and emergencies with effective solutions in an aging country. In fact, the data indicate that in Italy in 2040 there will be over 19 million elderly and 28 million chronic patients, with increases respectively of + 38.5% (+5.4 million elderly) and + 12% (+3 million chronic patients). To this, we must add the “suspended” Healthcare emergency: 46 million specialist visits, diagnostic tests, and 3 million fewer oncological screenings in 2020 compared to the previous year, which we will soon return to engage the NHS.

Therefore, digital health is the challenge to be mastered. The experiences achieved so far independently from the various territories have given rise to an infinite multiplication of platforms and projects with the result that Italy boasts a babel of software, devices and technologies. The fragility of digital has emerged and, to date, the inability to inform health care about oneself because digitalization is frequently insufficient or error-prone, generating more harm than benefit.

## Author Contributions

LP: conceptualization, methodology, validation, investigation, writing—original draft, writing—review and editing, and supervision. VM: data curation, investigation, writing—original draft, and writing—review and editing. GP, SS, CB, BB, and MG: writing—review and editing, supervision, and funding acquisition. All authors contributed to the article and approved the submitted version.

## Funding

This study was funded by a project of the San Paolo banking foundation, codified as C347A – SELF PORTRAIT SAN PAOLO.

## Conflict of Interest

The authors declare that the research was conducted in the absence of any commercial or financial relationships that could be construed as a potential conflict of interest.

## Publisher's Note

All claims expressed in this article are solely those of the authors and do not necessarily represent those of their affiliated organizations, or those of the publisher, the editors and the reviewers. Any product that may be evaluated in this article, or claim that may be made by its manufacturer, is not guaranteed or endorsed by the publisher.
